# Mapping the Memory Structure of High-Knowledge Students: A Longitudinal Semantic Network Analysis

**DOI:** 10.3390/jintelligence12060056

**Published:** 2024-05-31

**Authors:** Simone A. Luchini, Shuyao Wang, Yoed N. Kenett, Roger E. Beaty

**Affiliations:** 1 Department of Psychology, Pennsylvania State University, University Park, PA 16802, USA; ginawsywork@gmail.com; 2 Department of Psychological and Brain Sciences, Drexel University, Philadelphia, PA 19104, USA; 3 Faculty of Data and Decision Sciences, Technion—Israel Institute of Technology, Haifa 320003, Israel; yoedk@technion.ac.il

**Keywords:** cognitive network science, educational assessment, expertise, knowledge, semantic memory, undergraduate education

## Abstract

Standard learning assessments like multiple-choice questions measure what students know but not how their knowledge is organized. Recent advances in cognitive network science provide quantitative tools for modeling the structure of semantic memory, revealing key learning mechanisms. In two studies, we examined the semantic memory networks of undergraduate students enrolled in an introductory psychology course. In Study 1, we administered a cumulative multiple-choice test of psychology knowledge, the Intro Psych Test, at the end of the course. To estimate semantic memory networks, we administered two verbal fluency tasks: domain-specific fluency (naming psychology concepts) and domain-general fluency (naming animals). Based on their performance on the Intro Psych Test, we categorized students into a high-knowledge or low-knowledge group, and compared their semantic memory networks. Study 1 (N = 213) found that the high-knowledge group had semantic memory networks that were more clustered, with shorter distances between concepts—across both the domain-specific (psychology) and domain-general (animal) categories—compared to the low-knowledge group. In Study 2 (N = 145), we replicated and extended these findings in a longitudinal study, collecting data near the start and end of the semester. In addition to replicating Study 1, we found the semantic memory networks of high-knowledge students became more interconnected over time, across both domain-general and domain-specific categories. These findings suggest that successful learners show a distinct semantic memory organization—characterized by high connectivity and short path distances between concepts—highlighting the utility of cognitive network science for studying variation in student learning.

## 1. Introduction

Psychologists have long been interested in studying the relationship between learning and memory, a link that is of considerable importance for informing modern educational practices (Anderson 2000). To evaluate student learning, educators often employ assessments such as multiple-choice quizzes or short-answer questions (Becker and Watts 2001). Despite their popularity, such assessments can only evaluate what students know on a surface level. To provide a deeper understanding of student learning, researchers have recently employed methods from cognitive network science that can model (latent) knowledge structures. Network science quantifies the relationships between units in a complex system—such as words in a semantic memory network—providing powerful tools for understanding how students represent and retrieve knowledge to facilitate successful learning and academic performance (Nesbit and Adesope 2006; Siew 2020). Previous cross-sectional research has found that older students have different knowledge structures compared to younger students across a variety of academic subjects (Siew and Guru 2022). To date, no study has employed network science to compare the knowledge structures of more and less knowledgeable students taking the same academic course. In the present research, we address this gap by examining the knowledge structures of students with higher levels of course knowledge, investigating whether their representation of concepts differs from students who learn less course knowledge.

Assessing student learning is of vital importance in education, as it provides a means to identify gaps in knowledge, provide directed feedback, as well as determine academic achievement (Suskie 2018). Since the popularization of pen-and-paper examinations in the 1920s, student learning has often been evaluated in terms of raw information retention on multiple-choice quizzes (Stiggins 1991). Despite certain advantages, such as quick grading, such assessments have been criticized for their poor effectiveness at measuring students’ understanding of a topic (Biggs 1973; Entwistle and Entwistle 1992). Other assessments which favor concept understanding come then in the form of constructed responses such as short-answer questions (Martinez 1999). Although constructed responses allow for a more nuanced measurement of student learning, they carry their own downsides such as long grading times (Simkin and Kuechler 2005). Of note, neither multiple-choice nor constructed responses are able to tap into the hidden mental structures formed by learned concepts (Siew and Guru 2022). These memory structures have been shown to allow for a unique evaluation of a student’s understanding of concepts and problems within a domain, distinguishing more from less experienced students, and may ultimately serve as a valid complementary tool to traditional learning assessments (Chi et al. 1981; Siew 2019).

A common way of measuring student knowledge structures has been concept maps—diagrams representing the relationships shared by concepts or ideas (Novak 2010; Novak and Cañas 2007). Concept maps are typically evaluated in terms of their visual properties, by judging the unique shape of each map and drawing qualitative conclusions about the memory structure that they reflect. In these terms, more experienced students tend to draw concept maps that are more “net-like”, with more connections between concepts, than the more “chain-like” concept maps drawn by less experienced students (Kinchin et al. 2000; Lavigne 2005). These kinds of conclusions have been regarded to be distinct from those allowed by typical educational assessments, given that concept maps may expose information on the nature of learned concepts, such as the relationships shared between them in long-term memory (Siew and Guru 2022). Concept maps have also proven to be a more effective tool than grades for measuring subject knowledge in students in low-income and culturally diverse schools (Maker and Zimmerman 2020). However, a major challenge of using concept maps in education or research is quantifying their structural properties so that learning may be clearly measured and compared across students (Rittle-Johnson and Schneider 2015; Ruiz-Primo and Shavelson 1996).

One potential solution has emerged from the use of network science to analyze concept maps as mathematical graphs (Koponen and Nousiainen 2014; Koponen and Pehkonen 2010; Siew 2019). Mathematical graph theory involves the representation of complex systems as graphs or networks (e.g., Börner et al. 2008; Newman et al. 2006). Networks are made up of nodes (e.g., an idea or concept) which are connected to each other via edges (e.g., the similarity between two edges). In the study of human cognition, there has been a growing interest in using network science methodologies (Baronchelli et al. 2013; Siew et al. 2019). This trend is mainly due to the availability of quantitative tools for modelling semantic memory—consistent with longstanding theoretical accounts which posit that semantic memory is structured as a network (Collins and Loftus 1975; Smith et al. 1974). For instance, network science allowed researchers to demonstrate that a Montessori school curriculum, compared to a traditional one, promoted more “flexible” memory structures in children, with higher connectivity and shorter paths between concepts (Denervaud et al. 2021). Similar research has shown how creativity relates to second language learning, exhibited via more “flexible” semantic memory structures of the learned language (Kenett 2024). This structure is conducive to connecting concepts in semantic memory networks, and has previously been associated with higher cognitive abilities, including creative thinking (He et al. 2021; Kenett 2024).

Network science has also been employed for the quantitative analysis of concept maps drawn by university students enrolled in an introductory psychology course (Siew 2019). Concept maps were drawn based on topics covered in a psychology textbook chapter (i.e., neuroscience), which were included in a later quiz. Students who scored higher on the quiz also exhibited longer paths between concepts in their maps, indicating that higher content knowledge was associated with representing concepts further apart from each other. This finding may appear counterintuitive when considering that networks with shorter paths and higher clustering of nodes, also known as “small-world” networks, have consistently been associated with higher processing efficiency (He et al. 2021; Watts and Strogatz 1998), flexibility (Kenett et al. 2018), and creative thinking (Kenett 2024). However, higher fluid intelligence has been related to longer paths between concepts, alongside more compartmentalized semantic memory networks, that exhibit more discrete conceptual subcategories (e.g., types of animals in the animal category), suggesting that a well-structured semantic memory network may facilitate memory search and retrieval (Kenett 2024). Thus, a critical question for the current research is whether students’ effective learning is reflected in more structured or more flexible semantic memory networks.

Recently, Siew and Guru (2022) adopted the verbal fluency task—which involves generating words based on an initial prompt word—to model semantic memory networks of university and high school students. One version of this task, the animal fluency task, is widely used to measure domain-general semantic memory, i.e., general knowledge categories, as the animal category has been found to be the most stable across cultures and languages (Ardila et al. 2006). Verbal fluency data are typically analyzed via group-based networks that require the aggregation of participants into discrete groups (Christensen and Kenett 2023; Zemla and Austerweil 2018). Siew and Guru (2022) compared both domain-general (animal, fruit) and domain-specific (psychology, biology) semantic memory networks of university students and novice high-school students. The authors found that university students had memory structures that were more small-world across both domain-general and domain-specific categories compared to novice high-school students, supporting the view that domain knowledge is linked with more flexible/less structured memory structures.

### The Present Research

The investigation by Siew and Guru (2022) shed light on the relationship between a student’s learning and knowledge structure. However, comparing groups of different ages can make it difficult to disentangle whether group differences are related to domain knowledge or cognitive development, i.e., whether students differ in their knowledge structures due to learning or age-related changes in the semantic system. Moreover, measuring students at a single timepoint makes it hard to disentangle learning from other factors that may influence domain expertise, such as individual differences in cognitive ability. The present paper thus aims to build upon these findings by comparing age-matched students with varying levels of domain-specific expertise (Study 1). Further, we test students at two separate timepoints in the academic semester and compare whether any changes in knowledge structure are associated with learning (Study 2).

## 2. Study 1

In Study 1, we aimed to test whether learning was associated with structural differences in the semantic memory of students. To measure domain-general and domain-specific memory structures, we employed the verbal fluency task, which is commonly used to estimate semantic memory networks (Christensen and Kenett 2023). Undergraduate students were separated into a high-knowledge or low-knowledge group based on their scores on a cumulative psychology test at the end of the course. We hypothesized that higher psychology knowledge would be related to more interconnected semantic memory networks for psychology (i.e., domain-specific networks), with psychology concepts being more richly connected to each-other, consistent with past work using different experimental designs (e.g., Kinchin et al. 2000; Lavigne 2005; Siew and Guru 2022). Given past work linking expertise and general semantic memory structure, we further expected that higher psychology knowledge would lead to domain-general semantic memory networks that would be more interconnected and less modular (Siew and Guru 2022).

### 2.1. Materials and Methods

#### 2.1.1. Participants

A total of 267 (184 females; 79 males; 4 non-binary; M = 18.97 years, SD = 2.73 years) participants who were enrolled in an undergraduate introductory psychology class were recruited from The Pennsylvania State University (PSU). Participants were tested, near the end of the academic semester, via an online battery of cognitive tasks lasting 1 h. Beyond the tasks reported in the following analyses, the battery also included a series of creativity tasks that were completed after the verbal fluency tasks and the Intro Psych Test. The study was approved by the PSU Institutional Review Board.

#### 2.1.2. Materials

*Animal Fluency Task*. The animal fluency task was administered to estimate domain-general semantic networks—the most commonly used task for estimating group-based semantic memory networks (Christensen and Kenett 2023). The duration of the animal fluency task was three minutes (Ardila et al. 2006). During this time, participants were required to generate (type) as many animal names as they could, and to continue responding until the time was over. The task was performed with a computer keyboard, using the Enter key to submit responses.

*Psychology Fluency Task*. A psychology fluency task was administered to estimate domain-specific semantic networks, consistent with past work (Siew and Guru 2022). The task was administered the same way as the animal fluency task, except that participants were required to generate words associated with psychology for the duration of the task, following Siew and Guru (2022).

*Intro Psych Test*. A multiple-choice test was constructed to assess psychology knowledge (see Appendix A). The senior author coordinated with the course instructor, who shared the syllabus and study guides listing the topics covered in the course. The test was administered at the end of the semester to ensure students had been exposed to all topics. A total of 37 questions were developed based on an introductory psychology textbook, including the following topics: biopsychology, development, learning, memory, perception, and social psychology. After completing the test, students were asked to self-report their current grade, using a 9-point Likert scale (i.e., 1 = D; 2 = C−; 3 = C; 4 = C+; 5 = B−; 6 = B; 7 = B+; 8 = A−; 9 = A); students could skip the question if they did not know their current grade. The purpose of reporting grades was to validate our new Intro Psych Test.

#### 2.1.3. Group Construction

We constructed group-based semantic memory networks using the psychology (domain-specific) and animal (domain-general) fluency responses, which required aggregating participants into groups (Christensen and Kenett 2023). We separated participants into two groups via a median split based on their performance on the Intro Psych Test. We removed participants if they generated less than 3 responses to any of the fluency tasks (N = 2). Participants were also removed at the median number of correct responses (N = 64) so that the groups would be well-defined, ensuring that “boundary” cases would be addressed (i.e., participants with median scores belong to neither the “high” or “low” group; Irwin and McClelland 2003). We thus retained a high-psychology-knowledge group (N = 114; 84 females; 28 males; 2 non-binary; M = 19.1 years, SD = 3.06 years) and a low-psychology-knowledge group (N = 87; 61 females; 24 males; 2 non-binary; M = 18.7 years, SD = 0.83 years) for a comparison of their semantic memory networks.

#### 2.1.4. Semantic Memory Network Estimation

The SemNA pipeline (Christensen and Kenett 2023)—an open access pipeline in R for the estimation and analysis of semantic memory networks from semantic fluency data—was adopted for preprocessing and analysis purposes, using the following steps:

*Preprocessing*. Automatic preprocessing of the semantic fluency data was conducted via two R packages: SemNetDictionaries (version 0.2.0) and SemNetCleaner (version 1.3.4; Christensen and Kenett 2023). The entire preprocessing procedure was run separately for the animal fluency and the psychology fluency data, taking the same steps for both datasets. First, within-participant repetitions (i.e., duplicate responses) and non-category members (for the animal fluency task: e.g., dragon, ant colony, moon) were removed from the data. Several other potential issues in the responses were then addressed, such as spelling errors, compound responses, root word variations, and continuous strings. Next, manual spell-checking was run, by psychology experts, over words that were not recognized by the software, which were then corrected accordingly to standard English.

A binary response matrix was then generated by transforming the cleaned data, with each unique response given across participants as columns, and individual participants as rows. The frequency of within-participant response occurrence was used to generate the content of the response matrix, with values either 1 (i.e., participant *i* generated exemplar *j*) or 0 (i.e., participant *i* did not generate exemplar *j*). Response exemplars included in the response matrix were limited to those that were provided by at least two participants in the overall sample, as this has been shown to allow for better control of confounding factors (e.g., differences in the number of nodes and edges between groups; Christensen and Kenett 2023). To further control for the confounding effect of including a different number of nodes between groups (Van Wijk et al. 2010), responses in the binary matrices were then equated across groups, retaining for each group only those responses that were provided by the other groups. To sum up, all comparisons of semantic memory network structure included in the present study consider only the differences in the organization of the same nodes between the semantic memory networks.

*Network Construction*. We conducted two network analyses between the low- and high-psychology-knowledge groups, separately for the psychology and animal fluency data. Both network analyses were run the same. Association profiles were computed between the fluency responses using the SemNeT (version 1.4.4) package (Christensen and Kenett 2023) in R (version 4.2.0) using R studio (version 2022.02.3). Network edges were calculated via the cosine similarity function in the SemNeT package which generates an n × n adjacency matrix (i.e., associations between each response) for each group (Christensen and Kenett 2023). Cosine similarity estimates the co-occurrence probability of two words by calculating the angle between two-word vectors—a commonly used technique in latent semantic analysis of text corpora (Landauer and Dumais 1997) and related methods of semantic distance computation (Beaty and Johnson 2021). Cosine similarity values range from 0 to 1; value of 1 represents two words that always co-occur, while 0 represents two words that never co-occur.

Using the SemNeT package, we applied the triangulated maximally filtered graph (TMFG; Christensen and Kenett 2023; Massara et al. 2016) to the adjacency matrix of each group. TMFG captures only the most reliable relations within the cosine-determined networks—preventing spurious associations from being retained in the final networks (Christensen and Kenett 2023)—by applying a structural constraint on the association matrix, restricting the number of edges which can be retained in the final networks.

*Network Analysis*. Three global network metrics were computed for each network, namely the clustering coefficient (CC), average shortest path length (ASPL), and modularity (Q). The CC of a network is a measure of connectivity, calculated as the extent to which two neighbors of a given node will themselves be neighbors. Higher CC values are associated with a more interconnected semantic memory network (Siew et al. 2019). The ASPL denotes the mean shortest number of edges required to traverse between any two nodes. The magnitude of the ASPL between any two nodes thus refers to the average relatedness of any two concepts within the network (Kenett et al. 2017; Kumar et al. 2020). Finally, Q measures network segregation, calculated as the extent to which a network possesses dense connections within sub-networks and between sub-networks. A higher Q is thus reflective of a higher degree of distinct sub-communities within the network (Fortunato 2010).

Our network analysis compared the network metrics (CC, ASPL, and Q) from the high- and low-psychology-knowledge groups against randomly generated networks. In accordance with established procedures when comparing group-based networks (Christensen and Kenett 2023), we employed a case-wise bootstrap analysis (Efron 1979) to analyze any differences in the network structure between-groups. As group-based calculations of network metrics only provide a single value per group and thus cannot be directly compared, bootstrapping serves as a test of significance for the network comparisons. The SemNeT package in R was employed to run the bootstrapping (Christensen and Kenett 2023), with 1000 iterations. Networks for the resampled groups were generated separately for each network, using with-replacement bootstrapping (Bertail 1997). Network measures (CC, ASPL, and Q) were then calculated for each resampled group’s network and the two networks were compared by conducting an independent-samples *t*-test analysis for each network metric.

#### 2.1.5. Procedure

Study 1 was conducted online through Pavlovia (https://pavlovia.org/ (accessed on 1 February 2024)) and completed by participants on their personal computers. All participants first completed the verbal fluency tasks (psychology and animal), counterbalanced in their order of presentation, and later completed the Intro Psych Test. At the end of the study, participants were asked for self-reported grades and demographic information.

### 2.2. Results

#### 2.2.1. Fluency and Descriptives

First, we tested whether any group differences exist between fluency scores (i.e., number of responses) on the psychology fluency and the animal fluency tasks, separately (Table 1). Regarding psychology fluency, the low-psychology-knowledge (M = 9.7, SD = 4.1) and high-psychology-knowledge (M = 10.2, SD = 3.9) groups were not significantly different, *t*(199) = 0.78, *p* = .44, *η*^2^ = 0.003, 95% CI [−0.68, 1.56]. Similarly, for animal fluency, we found no difference between the low-psychology-knowledge (M = 16.4, SD = 5.6) and high-psychology-knowledge (M = 17.4, SD = 4.6) groups, *t*(199) = 1.51, *p* = .13, *η*^2^ = 0.011, 95% CI [−0.33, 2.49], indicating comparable fluency performance between the two groups.

Next, we aimed to validate the Intro Psych Test with respect to self-reported course grades. We thus computed a Pearson correlation analysis between test performance and self-reported grades. Due to the high positive skew of the self-reported grades, the values were log-transformed before any analysis. We found a moderate positive linear relationship between test performance and self-reported grades, r = 0.30, *p* < .001, 95% CI [0.17, 0.42], indicating that students who performed better on the test tended to perform better in the course. For exploratory purposes, we also computed correlations between test performance and verbal fluency, finding no significant associations (Table 2): students who performed better on the test did not produce more psychology concepts or animal names on the fluency tasks.

#### 2.2.2. Semantic Memory Networks

**Figure 1:**
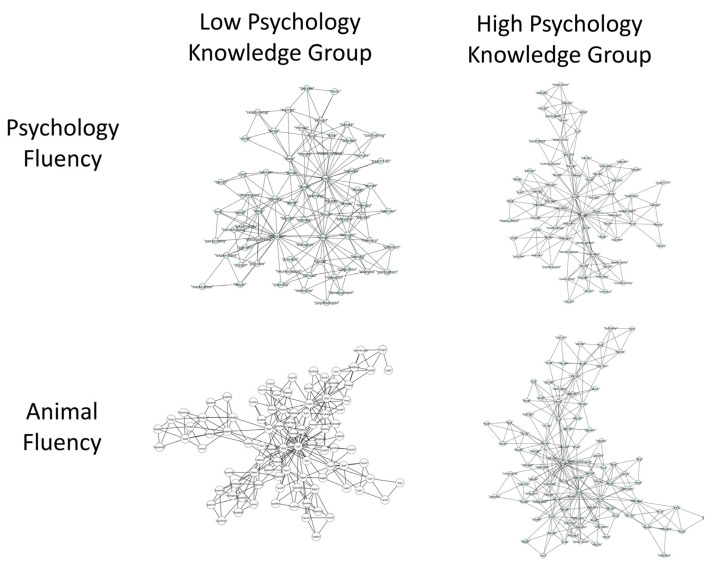
A 2D visualization of the psychology and animal semantic memory networks of individuals with high and low psychology knowledge. Note. Circles represent nodes (i.e., concepts) which are connected by edges based on the strength of the semantic associations between concepts in each group.

We next analyzed the semantic memory networks for the low- and high-psychology-knowledge groups, separately for the psychology and animal fluency tasks. This led to psychology semantic memory networks with 72 nodes and 205 edges, an average degree of 5.69, density of 0.08, and efficiency of 0.41. The animal semantic memory networks were composed of 96 nodes and 280 edges, an average degree of 5.83, density of 0.06, and efficiency of 0.40. Networks were visualized via Cytoscape 3.9.1 (Figure 1; Shannon et al. 2003), by generating 2D representations of unweighted and undirected networks, in which circles represent concepts and lines represent the links between concepts.

We tested whether the semantic memory networks of the low- and high-psychology-knowledge groups were significantly different from randomly generated networks, matched by the number of nodes and edges. This random network analysis revealed that for both psychology and animal fluency semantic memory networks, across both groups and for all network metrics (CC, ASPL, and Q), the empirically generated semantic networks were significantly different from randomly generated networks (all *p*s < .001).

**Figure 2:**
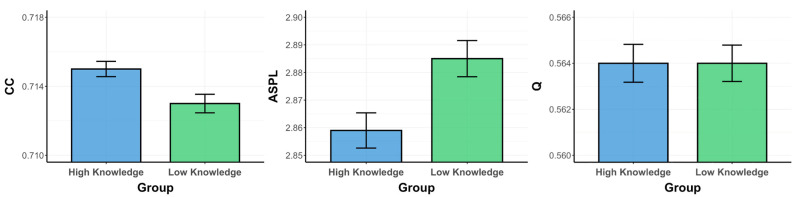
Psychology fluency networks metrics (CC/ASPL/Q) for psychology knowledge groups (High/Low). Note. Bootstrapping was run over 1000 iterations. Means of each group are presented for all network parameters. ASPL, average shortest path length; CC, clustering coefficient; Q, modularity.

**Figure 3:**
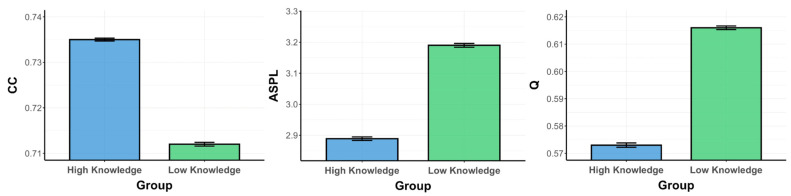
Animal fluency networks metrics (CC/ASPL/Q) for psychology knowledge groups (High/Low). Note. Bootstrapping was run over 1000 iterations. Means of each group are presented for all network parameters. ASPL, average shortest path length; CC, clustering coefficient; Q, modularity.

Critically, we then compared whether the low- and high-psychology-knowledge groups were significantly different from each other in the structure of their semantic memory networks for the psychology (Figure 2) and animal (Figure 3) domains, via the bootstrapping approach. We analyzed the data through a series of independent-samples *t*-tests, as well as by estimating Bayes factors using the Bayesian Information Criteria (Wagenmakers 2007). Bayes factors compare the likelihood of the data being explained by the alternate hypothesis, as opposed to the null hypothesis. In these terms, a Bayes factor of 3 is roughly equivalent to a *p* value of .05 in support of the alternate hypothesis (Wetzels et al. 2011).

*Psychology Fluency Networks.* For the psychology semantic memory networks, an independent-samples *t*-test revealed that the high-psychology-knowledge group exhibited a significantly higher CC (M = 0.715, SD = 0.014) than the low-psychology-knowledge group (M = 0.713, SD = 0.017), *t*(1998) =  2.85, *p* = .004, *d* = 0.13, 95% CI [0.001, 0.003]. Although trending towards support for the alternate hypothesis, the Bayes factor failed to meet our significance threshold of 3 (BF_10_ = 2.768). Further, the high-psychology-knowledge group exhibited a shorter ASPL (M = 2.859, SD = 0.202) than the low-psychology-knowledge group (M = 2.885, SD = 0.207), *t*(1998) = −2.84, *p* = .005, *d* = 0.13, 95% CI [−0.040, −0.008]. Although trending towards the alternate hypothesis, the Bayes factor again failed to meet our significance threshold (BF_10_ = 2.707). Lastly, the comparison for Q revealed that the high-psychology-knowledge group (M = 0.564, SD = 0.026) did not significantly differ from the low-psychology-knowledge group (M = 0.564, SD = 0.025), *t*(1998) =  0.04, *p* = .966, *d* = 0.002, 95% CI [−0.002, 0.002], as confirmed by the Bayes factor (BF_10_ = 0.05). Altogether, compared to the low-psychology-knowledge group, the semantic memory network of the high-psychology-knowledge group was significantly more connected (higher CC) and possessed shorter average paths (lower ASPL); however, the networks were similar in terms of communities (Q).

*Animal Fluency Networks.* For the animal semantic memory networks, an independent-sample *t*-test revealed that the high-psychology-knowledge group exhibited a significantly higher CC (M = 0.735, SD = 0.01) than the low-psychology-knowledge group (M = 0.712, SD = 0.013), *t*(1998) =  43.99, *p* < .001, *d* = 1.97, 95% CI [0.022, 0.024], BF_10_ = 6 × 10^291^. Further, the high-psychology-knowledge group exhibited a shorter ASPL (M = 2.889, SD = 0.18) than the low-psychology-knowledge group (M = 3.19, SD = 0.192), *t*(1998) = −36.24, *p* <  .001, *d* = 1.62, 95% CI [−0.318, −0.285], as confirmed by a Bayesian independent-samples *t*-test (BF10 = 1.5 × 10217). Lastly, the high-psychology-knowledge group exhibited a significantly lower Q (M = 0.573, SD = 0.025) than the low-psychology-knowledge group (M = 0.616, SD = 0.021), *t*(1998) =  −41.4, *p* < .001, *d* = 1.85, 95% CI [−0.044, −0.040], BF_10_ = 4.3 × 10^266^. Taken together, compared to the low-psychology-knowledge group, the semantic memory network of the high-psychology-knowledge group was significantly more connected (higher CC), with shorter average paths (lower ASPL) and fewer communities (lower Q).

### 2.3. Discussion

Evidence from past research indicates that more experienced university students possess a more small-world (i.e., higher clustering and shorter paths between concepts) semantic memory structure than less experienced high-school students (Siew and Guru 2022). However, given the confounding effect of age, the link between learning and semantic memory structure remains unclear. Study 1 addressed this limitation, by comparing age-matched groups of university students with low and high psychology knowledge. Students in the high-psychology-knowledge group were found to possess more small-world semantic memory networks for both the animal and psychology domains, although findings for the domain-specific network were found to be non-significant in accordance with the Bayes factors. This finding is generally consistent with the work from Siew and Guru (2022), pointing to a link between learning and more efficient semantic memory structures. However, for the domain-specific networks, we observed a non-significant effect with regard to Q, a measure of network communities. We speculate that this inconsistency relates to the effect of age on Q, with older individuals displaying more modular semantic memory structures (Cosgrove et al. 2023).

## 3. Study 2

In Study 1, we observed how the semantic memory structure of students enrolled in an introductory psychology course depended on their learning. In Study 2, we sought to confirm this finding, by including a longitudinal component in our measurements. This longitudinal approach allowed us to study—for the first time—how semantic networks change over time in students who learn more and have less course knowledge. Further, we administered a secondary multiple-choice psychology assessment, the psychology knowledge test (PsyKT). The PsyKT was taken from Kunina et al. (2007) and was included to determine the construct validity of our Intro Psych Test.

### 3.1. Materials and Methods

#### 3.1.1. Participants

We recruited an initial sample of 160 participants to participate in a two-session study. Our final sample size, given a retention rate of 91%, was of 145 participants (128 females; 16 males; 1 non-binary; M = 18.42 years, SD = 0.78 years) enrolled in an undergraduate introductory psychology class at PSU. Testing was conducted at two timepoints, once at the start of the academic semester (i.e., timepoint 1; T1) and again near the end (i.e., timepoint 2; T2). Participants completed an online battery of cognitive tasks lasting 1 h at each timepoint. A series of creative tasks and a language learning task were included at both timepoints. These tasks were performed after the verbal fluency tasks, the Intro Psych Test, and the Psychology Knowledge Test (PsyKT), and were not analyzed for the purposes of this study. The study was approved by the PSU IRB.

#### 3.1.2. Materials

*PsyKT*. In addition to the Intro Psych Test from Study 1 (see Appendix A), we administered a second, established assessment of psychological knowledge, the PsyKT, to test the construct validity of our Intro Psych Test. The assessment was extracted from a previous study which extensively validated its use in research with undergraduate psychology students (Kunina et al. 2007). The assessment contains 50 multiple choice questions on a variety of topics which fall within the umbrella of psychology. The assessment was originally devised in German, so it was translated into English for the purposes of this study.

#### 3.1.3. Group Construction

Based on their performance on the Intro Psych Test completed at T1, participants were separated into two groups via a median split. After removing participants at the median (N = 11), we retained a high-psychology-knowledge (N = 72; 60 females; 11 males; 1 non-binary; M = 18.47 years, SD = 0.92 years) and a low-psychology-knowledge group (N = 62; 57 females; 5 males; M = 18.34 years, SD = 0.57 years). Group-based semantic memory networks were then constructed separately for fluency responses collected at T1 and T2, for both psychology and animal fluency data, leading to 4 semantic memory networks being generated from each fluency task (high-knowledge T1/high-knowledge T2/low-knowledge T1/low-knowledge T2).

#### 3.1.4. Semantic Memory Network Estimation

Like in Study 1, we followed the SemNA pipeline for preprocessing and analysis of networks (Christensen and Kenett 2023). Statistical analysis also followed a similar procedure, with the exception of two sets of ANOVAs, run separately for psychology and animal networks. All ANOVAs included knowledge (high/low) and timepoint (T1/T2) as predictor variables, and included either CC, ASPL, or Q as predicted variables.

#### 3.1.5. Procedure

Online data collection was conducted through Pavlovia (https://pavlovia.org/ (accessed on 1 February 2024)) and completed on the participants’ personal computers. Participants first completed the verbal fluency tasks (psychology and animal), counterbalanced for order of presentation, before completing our Intro Psych Test and the PsyKT. Finally, participants responded to a series of questions relating to self-reported grades and demographics.

### 3.2. Results

#### 3.2.1. Fluency and Descriptives

We began by testing whether any differences in fluency existed between knowledge groups at any timepoint, separately analyzing the psychology fluency and the animal fluency tasks. For psychology fluency collected at T1, the low-psychology-knowledge (M = 11.9, SD = 3.7) and high-psychology-knowledge (M = 12.2, SD = 3.2) groups were not significantly different, *t*(127) = 0.514, *p* = .608, *d* = 0.002, 95% CI [−1.53, 0.9]. The same was true at T2, where the low-psychology-knowledge (M = 13.3, SD = 3.8) and high-psychology-knowledge (M = 13.5, SD = 4.1) groups were not significantly different in their psychology fluency, *t*(126) = 0.285, *p* = .776, *d* = 0.001, 95% CI [−1.18, 1.58]. For animal fluency, at T1, we observed no significant difference between the low-psychology-knowledge (M = 18.6, SD = 4) and high-psychology-knowledge (M = 19.3, SD = 3.6) groups, *t*(126) = 1.122, *p* = .26, *d* = 0.01, 95% CI [−2.1, 0.58]. For T2, there was also no difference in animal fluency between the low-psychology-knowledge (M = 19.2, SD = 3.5) and high-psychology-knowledge (M = 20, SD = 3.8) groups, *t*(125) = 1.227, *p* = .22, *d* = 0.01, 95% CI [−2.1, 0.49]. The results replicate Study 1, indicating no verbal fluency differences between the two groups for the domain-specific and domain-general categories used to estimate semantic memory networks. 

We then tested whether any fluency differences existed between timepoints, for any group, separately for psychology and animal fluency. For the high-psychology-knowledge group, we observed no significant difference in psychology fluency between T1 (M = 12.2, SD = 3.2) and T2 (M = 13.3, SD = 3.8), *t*(139) = 1.728, *p* = .09, *d* = 0.021, 95% CI [−0.15, 2.21]. We then observed a significant difference in psychology fluency between T1 (M = 11.9, SD = 3.7) and T2 (M = 13.5, SD = 4.1) for the low-psychology-knowledge group, *t*(114) = 2.130, *p* = .04, *d* = 0.04, 95% CI [0.11, 2.98]. For animal fluency, instead, we observed no significant difference between T1 (M = 19.3, SD = 3.6) and T2 (M = 20, SD = 3.8) for the high-psychology-knowledge group, *t*(136) = 1.035, *p* = .03, *d* = 0.008, 95% CI [−0.6, 1.91]. For the low-psychology-knowledge group there was also no difference in animal fluency between T1 (M = 18.6, SD = 4) and T2 (M = 19.2, SD = 3.5), *t*(115) = 0.879, *p* = .38, *d* = 0.007, 95% CI [−0.77, 2]. Thus, verbal fluency remained mostly stable over time, with the exception of the low-knowledge group showing a slight increase in psychology fluency from T1 to T2.

Next, we validated the Intro Psych Test with the self-reported course grades, and the PsyKT. Due to a high positive skew in the self-reported grades, log-transformation was applied before any analysis, like Study 1. We thus computed a Pearson correlation between performance on the Intro Psych Test at T1 and self-reported grades, finding a moderate correlation, *r* = 0.27, *p* = .001. We also found a moderate positive linear relationship between test performance at T2 and grades, *r* = 0.37, *p* < .001, indicating that students with better outcomes on the test, at the beginning or end of the course, tended to perform better in the course overall. We then tested whether the Intro Psych Test, separately for T1 and T2, correlated with the PsyKT. We observed moderate correlations between the two scales at both T1, *r* = 0.4, *p* < .001, and T2, *r* = 0.55, *p* < .001, providing evidence of the psychometric properties of our Intro Psych Test.

Then, we tested whether any learning had occurred between T1 and T2 by running paired-samples *t*-tests on the Intro Psych Test performance separately for the low-psychology-knowledge and high-psychology-knowledge groups. We found that the performance of the low-knowledge group increased between T1 (M = 15.1, SD = 2.5) and T2 (M = 19.8, SD = 4.7), *t*(58) = −8.82, *p* < .001, *d* = −1.25, 95% CI [−5.82, −3.67]. We similarly found that the performance of the high-knowledge group was better at T1 (M = 23, SD = 2.5) and T2 (M = 24.4, SD = 4.6), *t*(71) = −3.06, *p* = .003, *d* = −0.37, 95% CI [−2.27, −0.48]. Thus, as expected, students learned more about psychology concepts over time, and students with less initial knowledge learned the most.

We further explored our data by computing correlations between various descriptive variables (Table 3). Interestingly, we found positive relationships between performance on the Intro Psych Test and animal verbal fluency, indicating that students with better broad retrieval abilities performed better overall on our psychology multiple-choice test.

#### 3.2.2. Semantic Memory Networks

**Figure 4:**
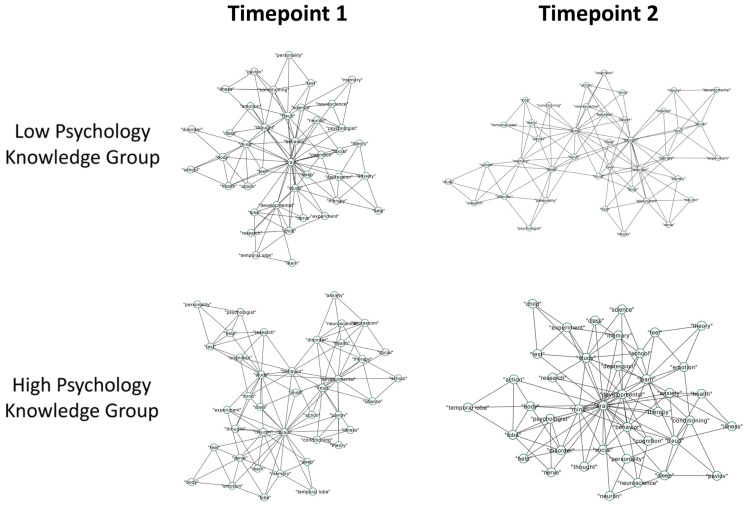
A 2D visualization of the psychology semantic memory networks of individuals with high and low psychology knowledge at timepoints 1 and 2. Note. Circles represent nodes (i.e., concepts) which are connected by edges based on the strength of the semantic associations between concepts in each group.

**Figure 5:**
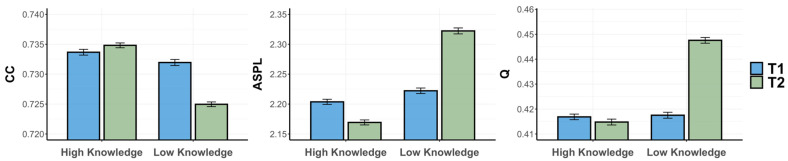
Psychology fluency networks metrics (CC/ASPL/Q), spanning knowledge (High/Low) and timepoint (T1/T2). Note. Bootstrapping was run over 1000 iterations. Means for each group and timepoint are presented for all network parameters. ASPL, average shortest path length; CC, clustering coefficient; Q, modularity.

**Figure 6:**
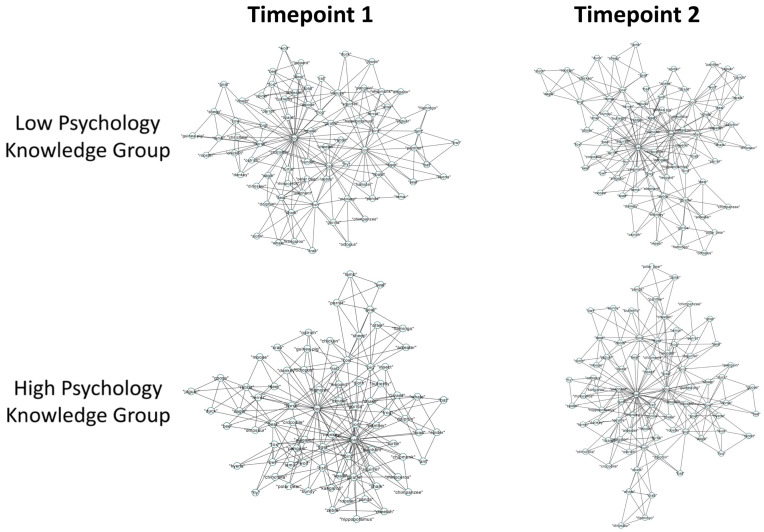
A 2D visualization of the animal semantic memory networks of individuals with high and low psychology knowledge at timepoints 1 and 2. Note. Circles represent nodes (i.e., concepts) which are connected by edges based on the strength of the semantic associations between concepts in each group.

**Figure 7:**
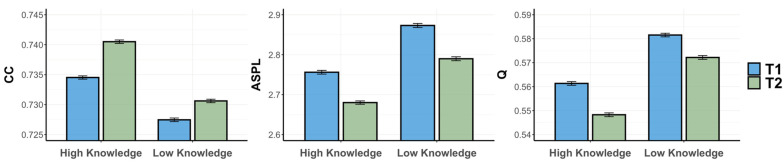
Animal fluency networks metrics (CC/ASPL/Q), spanning knowledge (High/Low) and timepoint (T1/T2). Note. Bootstrapping was run over 1000 iterations. Means for each group and timepoint are presented for all network parameters. ASPL, average shortest path length; CC, clustering coefficient; Q, modularity.

We analyzed the semantic memory networks for the low- and high-psychology-knowledge groups at T1 and T2, separately for the psychology and animal fluency tasks. Psychology semantic memory networks contained 40 nodes and 114 edges, an average degree of 5.7, density of 0.14, and efficiency of 0.5 (Figure 4 and Figure 5). Further, animal semantic memory networks had 80 nodes and 234 edges, an average degree of 5.85, density of 0.07, and efficiency of 0.46 (Figure 6 and Figure 7).

We tested whether semantic memory networks of the high- and low-psychology-knowledge groups, at both T1 and T2, were significantly different from random networks. This random network analysis revealed that the empirically generated networks for both groups, at both timepoints and for all network metrics (CC, ASPL, and Q), were significantly different from the randomly generated networks (all *p*s < .001). We then ran two sets of ANOVAs, separately for psychology and animal semantic memory networks, to investigate the effects of group and timepoint. Finally, we ran a series of pairwise comparisons via independent- and paired-samples *t*-tests, combined with calculations of Bayes factors, to investigate whether structural differences existed between any two networks.

*Psychology Fluency Networks.* First, we ran three separate ANOVAs for each of the network metrics of the psychology knowledge networks (CC, ASPL, and Q), with knowledge and timepoint as predictor variables (Figure 5). For our first ANOVA, we observed a significant interaction effect of knowledge and timepoint on CC, *F*(3996) = 81.968, *p* < .001, *η*^2^ = 0.02. We then found a significant main effect of knowledge on CC, *F*(3996) = 7.303, *p* = .007, *η*^2^ = 0.002, 95% CI [−0.007, −0.005], and a non-significant main effect of timepoint, *F*(3996) = 3.282, *p* = .07, *η*^2^ = 0.001, 95% CI [−0.004, −0.002]. Then, we ran a series of pairwise comparisons to investigate the source of the interaction. We first computed two paired-samples *t*-tests, separately for the high- and low-psychology-knowledge groups, to determine whether any changes in CC existed between T1 and T2. We further ran two Bayesian paired-samples *t*-tests to confirm our findings. This revealed that only the low-knowledge group displayed a significant decrease in CC from T1 to T2, *t*(999) = −11.25, *p* < .001, BF_10_ = 2 × 10^23^, while the CC for the high-knowledge group did not differ between T1 and T2, *t*(999) = −1.89, *p* = .059, BF_10_ = 0.3. We computed two independent-samples *t*-tests, separately for T1 and T2, to determine whether there were any differences in CC between the low- and high-psychology-knowledge groups. To confirm our findings, we also ran two Bayesian independent-samples *t*-tests. We found the high-knowledge group possessed a higher CC at T1, *t*(999) = 2.5, *p* = .01, although this was not supported by the Bayes factor (BF_10_ = 1). Further, we found that the high-knowledge group also possessed a higher CC at T2, *t*(999) = 18.3, *p* < .001, which was confirmed by the Bayes factor (BF_10_ = 3.3 × 10^60^).

Next, we ran an ANOVA with ASPL as a predicted variable, revealing a significant interaction effect of knowledge and timepoint, *F*(3996) = 221.827, *p* < .001, *η*^2^ = 0.05. We also observed the significant main effects of knowledge *F*(3996) = 8.378, *p* = .004, *η*^2^ = 0.002, 95% CI [0.077, 0.095], and timepoint, *F*(1999) = 29.063, *p* < .001, *η*^2^ = 0.007, 95% CI [0.024, 0.042], on ASPL. We next ran two paired-samples *t*-tests, for the high- and low-psychology-knowledge groups, to test any differences between T1 and T2. The low-knowledge group displayed a significant increase in ASPL from T1 to T2, *t*(999) = −18.33, *p* < .001, BF_10_ = 2.7 × 10^43^, while the high-knowledge group showed a decrease in ASPL, *t*(999) = 7.05, *p* < .001, BF_10_ = 6.4 × 10^5^. We then ran two independent-samples *t*-tests, for T1 and T2, to test for any differences between the low- and high-psychology-knowledge groups. The high-psychology-knowledge group was found to possess a lower ASPL at both T1, *t*(999) = −3.64, *p* < .001, BF_10_ = 3.7, and T2, *t*(999) = −28.25, *p* < .001, BF_10_ = 4.2 × 10^104^.

Finally, for our ANOVA with Q as a predicted variable, we observed a significant interaction effect of knowledge and timepoint, *F*(3996) = 191.896, *p* < .001, *η*^2^ = 0.05, and non-significant main effects of both knowledge, *F*(3996) = 0.167, *p* = .683, *η*^2^ < 0.001, 95% CI [0.014, 0.018], and of timepoint, *F*(3996) = 1.593, *p* = .207, *η*^2^ < 0.001, 95% CI [0.012, 0.016]. We ran paired-samples *t*-tests for the high- and low-psychology-knowledge groups to investigate any differences between T1 and T2. While the low-knowledge group displayed a significant increase in Q from T1 to T2, *t*(999) = −22.4, *p* < .001, BF_10_ = 4.4 × 10^64^, the high-knowledge group showed no difference between T1 and T2, *t*(999) = 1.61, *p* = .11, BF_10_ = 0.1. Finally, we ran independent-samples *t*-tests to test whether the low- and high-psychology-knowledge groups differed at either T1 or T2. The high-psychology-knowledge group was found to possess a lower Q only at T2, *t*(999) = −25.06, *p* < .001, BF_10_ = 7.2 × 10^76^, but not T1, *t*(999) = −0.53, *p* = .59, BF_10_ = 0.05. Thus, the low-knowledge group showed significantly reduced connectivity (lower CC) and longer average paths (higher ASPL) from T1 to T2, despite demonstrating improvements in learning. This is visually evidenced by the nodes growing further apart, as well as an increase in the number of isolated nodes from T1 to T2. In contrast, for the high-knowledge group, the average paths became shorter between T1 and T2. Further, for the high-knowledge group, connectivity remained higher and average paths shorter at both T1 and T2 when compared to the low-knowledge group. Visually, this can be observed in the increased closeness of nodes from T1 to T2, as well as a reduction in the number of isolated nodes from T1 to T2, for the high-knowledge group. For instance, looking at the central node of the high-knowledge network, there is a visually noticeable increase in the number of connections to the concept of “brain” between T1 and T2. In contrast, the network of the low-knowledge group displays a noticeable decrease in connections to this same node from T1 to T2, denoting a reduction in the clustering of the network.

*Animal Fluency Networks*. We then ran three ANOVAs for the network metrics (CC, ASPL, Q) of the animal knowledge networks for both groups across both time groups (Figure 7). We observed a significant interaction effect of knowledge and timepoint on CC, *F*(3996) = 23.902, *p* < .001, *η*^2^ = 0.006. We also found significant main effects of knowledge, *F*(3996) =298.796, *p* < .001, *η*^2^ = 0.07, 95% CI [−0.009, −0.007], and timepoint, *F*(3996) = 213.998, *p* < .001, *η*^2^ = 0.05, 95% CI [0.004, 0.005], on CC. We then computed a series of paired- and independent-samples *t*-tests to investigate the effects. We first ran two paired-samples *t*-tests, and two Bayesian paired-samples *t*-tests, separately for the high- and low-psychology-knowledge groups, to determine whether there was any difference between T1 and T2. It was revealed that both the low-knowledge group, *t*(999) = −7.61, *p* < .001, BF_10_ = 7 × 10^10^, and high-knowledge group showed an increase in CC from T1 to T2, *t*(999) = −15.6, *p* < .001, BF_10_ = 2.7 × 10^44^. We then computed two independent-samples *t*-tests for T1 and T2 to determine whether there was any difference in the CC of the low- and high-psychology-knowledge groups. We further confirmed these findings by running two Bayesian independent-samples *t*-tests. The high-psychology-knowledge group was found to possess a higher CC, both at T1, *t*(999) = 17.7, *p* < .001, BF_10_ = 2.4 × 10^59^, and T2, *t*(999) = 24.1, *p* < .001, BF_10_ = 7.2 × 10^108^.

For ASPL, we observed a non-significant interaction effect of knowledge and timepoint, *F*(3996) = 0.596, *p* = .44, *η*^2^ < 0.001. We then observed the significant main effects of knowledge, *F*(3996) = 308.637, *p* < .001, *η*^2^ = 0.07, 95% CI [0.1, 0.12], and timepoint, *F*(3996) = 130.150, *p* < .001, *η*^2^ = 0.03, 95% CI [−0.09, −0.07], on ASPL. Next, we computed a series of paired- and independent-samples *t*-tests. We first ran two paired *t*-tests to determine whether there was any difference between T1 and T2 for the high- and low-psychology-knowledge groups. Both the low-knowledge group, *t*(999) = 12.49, *p* < .001, BF_10_ = 6.3 × 10^27^, and high-knowledge group showed a decrease in ASPL from T1 to T2, *t*(999) = 13.55, *p* < .001, BF_10_ = 3.3 × 10^29^. We next ran independent-samples *t*-tests for T1 and T2 to determine whether the low- and high-psychology-knowledge groups differ in their ASPL. The high-psychology-knowledge group had a shorter ASPL at T1, *t*(999) = −18.06, *p* < .001, BF_10_ = 2.3 × 10^57^, and T2, *t*(999) = −17.77, *p* < .001, BF_10_ = 1.1 × 10^57^.

Then, for Q, we observed a significant interaction effect of knowledge and timepoint, *F*(3996) = 5.777, *p* = .016, *η*^2^ = 0.001. Again, for Q, we observed the significant main effects of knowledge, *F*(3996) = 341.166, *p* < .001, *η*^2^ = 0.08, 95% CI [0.021, 0.024], and timepoint, *F*(3996) = 142.882, *p* < .001, *η*^2^ = 0.04, 95% CI [−0.013, −0.009]. We then ran pairwise comparisons between networks, starting with two paired *t*-tests to reveal any difference between T1 and T2 for the high- and low-psychology-knowledge groups. Both the low-knowledge group, *t*(999) = 9.2, *p* < .001, BF_10_ = 2 × 10^14^, and high-knowledge group, *t*(999) = 12.98, *p* < .001, BF_10_ = 2 × 10^28^, displayed a lower Q at T2 compared to T1. Finally, we ran independent-samples *t*-tests, separately for T1 and T2, to reveal whether the low- and high-psychology-knowledge groups differed in their Q. The high-psychology-knowledge group possessed a lower Q at T1, *t*(999) = −20.89, *p* < .001, BF_10_ = 7.4 × 10^69^, and T2, *t*(999) = −22.66, *p* < .001, BF_10_ = 4.2 × 10^87^. We thus revealed a similar effect of time for both the low- and high-knowledge groups, across all network metrics. Both groups demonstrated significantly increased connectivity (higher CC), shortened average paths (lower ASPL), and fewer communities (lower Q) from T1 to T2. This is visually evidenced by an increased closeness of nodes and a reduction in the number of isolated nodes from T1 to T2 for both groups.

### 3.3. Discussion

The goal for Study 2 was to replicate and extend Study 1 via a longitudinal investigation of student learning and memory structure. Study 2 directly replicated Study 1: when tested near the end of the academic semester, at T2, students with higher psychology knowledge possessed more small-world knowledge structures (i.e., higher clustering and shorter paths between concepts). Furthermore, longitudinal analysis showed that the semantic networks of high-knowledge students became even more interconnected over the course of the semester, leading to larger effect sizes at T2. Despite the low-knowledge students showing substantial learning over time, their networks became less interconnected, and thus less similar to high-knowledge students. These findings confirm past evidence indicating that learning is related to semantic memory structure, by demonstrating that learning is accompanied by structural reorganizations of semantic memory (Siew 2020; Siew and Guru 2022). Further, we provide evidence that students who possess more efficient semantic memory structures are more likely to succeed in a university-level course, as indicated by stronger learning and higher expected grades.

## 4. General Discussion

Typical educational assessments are commonly used by educators to measure student learning, but they can only evaluate surface-level knowledge (Siew and Guru 2022). To gain deeper insights into student learning, researchers have begun to examine how students organize knowledge using cognitive network science, which offers a viable, valid, and complementary approach to traditional educational assessments (Disessa and Sherin 1998; Siew 2020). In the present research, we used cognitive network science methods to model the knowledge organization of students who learned more and less in an introductory psychology course. In Study 1, students were only tested near the end of the academic semester, while in Study 2 they were tested both near the start (T1) and end (T2) of the semester. Students were separated into either a low- or a high-psychology-knowledge group based on their performance on a psychology multiple-choice test, the Intro Psych Test. We estimated domain-specific (psychology concepts) and domain-general (animal) semantic memory networks for each group using verbal fluency responses.

In Study 1, we found that the high-knowledge group exhibited a more small-world semantic memory structure—marked by shorter path distances and higher connectivity between concepts, for both domain-specific and domain-general networks—compared to the low-knowledge group. In Study 2, we directly replicated these findings and further revealed a dynamic interplay between network structure and learning. First, we found that the semantic memory networks of the high-knowledge group, both domain-specific and domain-general, were already more small-world at T1. This small-world memory structure of high-knowledge students was further emphasized at T2, both when compared to the low-knowledge group and to themselves at T1. These findings extend past research on the relationship between academic expertise and semantic memory structure (Nesbit and Adesope 2006; Siew 2020; Siew and Guru 2022), providing further evidence that semantic memory networks may be predictive of performance in educational contexts.

A key finding of Studies 1 and 2 was that the psychology semantic memory network for the high-knowledge group showed shorter paths between concepts than the low-knowledge group. Importantly, shorter path lengths have been found to facilitate relatedness judgments (Kenett et al. 2017; Kumar et al. 2020), as well as word retrieval and selection (Arbesman et al. 2010; Vitevitch et al. 2012). Hence, the knowledge structure of high-knowledge students may play a bottom-up, facilitatory role during memory retrieval (Siew 2020). This, in turn, would plausibly lead to better performance on other learning assessments, which strongly depend on recall and recognition processes, such as with the Intro Psych Test administered in this study. Our findings are consistent with the recent work of Siew and Guru (2022), finding that the networks of high-psychology-knowledge students were characterized by a shorter ASPL.

In both Studies 1 and 2, the domain-general/animal network mirrored the structure of the domain-specific/psychology network, similar to Siew and Guru (2022). This similarity cannot be directly accounted for by domain expertise, i.e., performance on the Intro Psych Test. One possibility is that students in the high-knowledge group had a cognitive advantage that predisposed them towards developing more efficient domain-specific memory structures, such as higher levels of pre-existing domain-general knowledge (i.e., crystallized intelligence) or stronger reasoning abilities that facilitate learning (i.e., fluid intelligence). This is supported by findings from Study 2, indicating that high-knowledge students possessed more small-world memory structures early in the semester, and these networks became even more small-world with learning. Both high fluid and crystallized intelligence have been shown to facilitate learning and academic achievement in an academic setting (e.g., Deary et al. 2007). While higher fluid intelligence has been linked to a more structured semantic memory network (Kenett 2024; Rastelli et al. 2020), crystallized intelligence has instead been linked with more flexible memory, such as that of high-psychology-knowledge students in our study (Li et al. 2024). Other cognitive abilities associated with crystallized intelligence, such as verbal creativity, have also been associated with less structured networks (He et al. 2021; Kenett 2024; Luchini et al. 2023), consistent with the present work. Further, prior work on language acquisition found that newly learned concepts are integrated in a network via a preferential attachment to more central nodes—those possessing a higher degree of connections (Steyvers and Tenenbaum 2005)—potentially benefiting students with more clustered semantic memory networks that may have more “hooks” to integrate new concepts.

Interestingly, in Study 1 we found no difference in the Q metric on the domain-specific/psychology networks, although this difference was present for the domain-general/animal network. This was partially replicated in Study 2, as we saw no difference between knowledge groups at T1 but found that low-knowledge students developed a more modular network at T2. Our general findings are only partly in line with Siew and Guru (2022), who reported higher levels of Q for both domain-general and domain-specific memory networks of high school students compared to college students. One possibility for this discrepancy might be that groups in the present study were matched on age, whilst in the Siew and Guru (2022) study they were generated by contrasting high-school and university students. Research on aging has shown that older adults tend to possess semantic memory structures that are more modular, possibly because of increasing vocabulary knowledge (Cosgrove et al. 2023). Curiously, the findings of Siew and Guru (2022) point toward more experienced, and older, students possessing less modular domain-general and domain-specific networks. It must be noted that the age difference between participants in the study by Cosgrove et al. (2023) was much larger than that in the study by Siew and Guru (2022), which only compared high-school and university students. It may thus be that the relationship between modularity and age is a non-linear one, meaning that modularity decreases when developing into young adulthood, before increasing again into older adulthood. It might then be that for Study 2, the increase in modularity for the domain-specific and domain-general networks of low-knowledge students is indicative of a deviation from typical developmental trends. Thus, the findings of Siew and Guru (2022) may, in part, be driven by an effect of age and vocabulary knowledge, beyond mere education.

Overall, the present findings extend past work exploring the use of memory structure assessments in the context of student knowledge evaluation (Chi et al. 1981; Siew 2019), providing the first longitudinal evidence that student learning is related to semantic memory network structure. Our findings are particularly relevant to educational settings, given the importance of measuring student learning (Suskie 2018), and the downsides of typical assessments such as multiple-choice and constructed questions (Biggs 1973; Entwistle and Entwistle 1992; Simkin and Kuechler 2005). Indeed, traditional assessments of learning cannot easily assess semantic memory structure (Siew and Guru 2022), which can provide unique insights into the learning of students (Chen and Poquet 2022). In the present research, we observe that predispositions in the semantic memory structure of psychology students are associated with learning outcomes across an academic semester. Indeed, students with more small-world memory structures had a better knowledge of psychology concepts, and reported higher expected grades in a psychology course, both at the start and end of the academic semester.

### Limitations and Future Directions

Despite the strengths of the current study, a few limitations should be mentioned. It is important to emphasize that the present work is correlational, leaving open the question of directionality. It remains unclear whether efficient learning engenders these characteristic memory structures associated with higher knowledge, and vice versa. It is also worth noting that semantic memory networks may also depend on executive abilities, meaning that what may appear to be a distinct memory structure could also be explainable by memory search processes (Siew et al. 2019). Moreover, we did not include measurements of fluid or crystallized intelligence in this study, which have been found to be strongly associated with academic performance (Deary et al. 2007; Soares et al. 2015) and semantic memory structure (Kenett 2024; Li et al. 2024). Further studies are therefore needed to determine whether fluid intelligence has any clear moderating effect between learning and semantic memory network restructuring.

Another limitation is the use of a group-based network estimation method. We adopted the verbal fluency task as it is currently the most common and easily replicable approach to estimate semantic memory networks (Zemla et al. 2020). Recent methodological advancements have been made in modeling individual-based semantic memory networks (Benedek et al. 2017; Morais et al. 2013; Wulff et al. 2022; Zemla and Austerweil 2018). These approaches do not require a dichotomization of the grouping variable, preventing issues that may arise from reduced granularity, such as loss of power or effect sizes (MacCallum et al. 2002), or overestimation of effect sizes (Conway et al. 2005; Preacher et al. 2005). It is possible that the present approach of dichotomizing the grouping variable may have led to an underestimation or overestimation of effects. Further, the approach of dichotomizing the grouping variable led to unequal sample sizes between the low- and high-psychology-knowledge groups, potentially affecting the results. Future studies are thus required to replicate the present work by employing larger sample sizes, as well as continuously estimated networks, particularly to determine the degree to which the ASPL of domain-specific semantic memory networks may depend on expertise. Further, the current dataset contains some individuals presenting with low performance on the verbal fluency, possibly due to sub-optimal performance. It is important that the present effects be replicated in an in-person sample of participants, thus ensuring that low-effort responding had no influence on the observed effects Despite these limitations, our findings offer important new insights into how knowledge is organized in the semantic memory of students with more or less course knowledge.

## 5. Conclusions

The present work replicates and extends past findings indicating that student knowledge can be accurately measured via network science approaches (Siew and Guru 2022). Crucially, this is the longitudinal first evidence that the memory structure of students enrolled in the same course can be quantitatively analyzed and related to their performance in the course. These findings inform further work investigating how memory structure relates to specific learning outcomes in students. This line of research may ultimately lead to the development of novel quantitative approaches for the measurement of student learning, the identification of gaps in learning, and the facilitation of teaching practices.

## Figures and Tables

**Table 1:** Descriptive statistics for the psychology and animal fluency task.

	Psychology Fluency Task				Animal Fluency Task			
	*N* (Average)		*N* (within)	*N* (between)	*N* (Average)		*N* (within)	*N* (between)
Group	*M (SD)*	Range			*M (SD)*	Range		
Low knowledge	9.7 (4.1)	3–22	307	156	16.4 (5.6)	3–29	362	177
High knowledge	10.2 (3.9)	3–24	359	208	17.4 (4.6)	3–31	354	185

Note. *N* (average) = the average number of responses in each group; *N* (within) = the total unique number of responses given by individuals within the group; *N* (between) = the total unique number of responses not given by the other groups.

**Table 2:** Descriptive statistics and correlations for the Intro Psych Test, self-reported grades, psychology verbal fluency and animal verbal fluency.

	*M*	*SD*	*NA*	Min, Max	1	2	3	4
Intro Psych Test	17.99	5.9	0	5, 32	1			
Self-Reported Grades	6.86	2.53	12	1, 10	**0.30**	1		
Psychology Fluency	9.98	3.98	0	3, 24	0.06	**0.15**	1	
Animal Fluency	16.98	5.05	0	3, 31	0.13	**0.14**	**0.43**	1

Note. NA = number of participants who refused to respond. Self-reported grades ranged in values from 1 to 9 and represent alphabetical grades. 10 = A+; 9 = A; 8 = A−; 7 = B+; 6 = B; 5 = B−; 4 = C+; 3 = C; 2 = C−; 1 = D. Statistically significant Pearson correlations are bolded (*p* < .05).

**Table 3:** Descriptive statistics and correlations for the Intro Psych Test at T1 and T2, the PsyKT, self-reported grades, psychology verbal fluency and animal verbal fluency.

	*M*	*SD*	*NA*	Min, Max	1	2	3	4	5	6	7
Intro Psych Test T1	19.32	4.54	0	9, 30							
Intro Psych Test T2	22.26	5.02	0	11, 34	**0.61**						
PsyKT	18.9	4.47	0	9, 29	**0.4**	**0.55**					
Self-Reported Grades	8.71	1.73	5	2, 10	**0.27**	**0.37**	**0.21**				
Psychology Fluency T1	12.14	3.83	0	4, 26	0.05	0.02	0.006	0.07			
Psychology Fluency T2	13.45	4.45	2	3, 29	−0.005	0.07	0.04	0.06	**0.56**		
Animal Fluency T1	18.89	4.32	0	6, 31	**0.18**	**0.24**	0.1	**0.19**	**0.43**	**0.23**	
Animal Fluency T2	19.16	4.51	1	6, 29	0.15	**0.17**	0.05	15	**0.47**	**0.49**	**0.54**

Note. NA = number of participants who refused to respond. Self-reported grades ranged in values from 1 to 9 and represent alphabetical grades. 10 = A+; 9 = A; 8 = A−; 7 = B+; 6 = B; 5 = B−; 4 = C+; 3 = C; 2 = C−; 1 = D. Statistically significant Pearson correlations are bolded (*p* < .05).

## Data Availability

All data used in this study is openly available on OSF: https://osf.io/gycs6/?view_only=3d101511551e407b9bd9d38151ba1608 (accessed on 1 May 2024).

## Appendix A

### Intro Psych Test

1. The cerebellum is primarily involved in directing
2. The frontal lobes primary role is in supporting
3. The hippocampus is a part of the
4. Neuroplasticity refers to how the nervous system can
5. The function of neurotransmitters in the nervous system is that of
6. The process of inputting information into the memory system is called
7. The memory store for personal life events is
8. Recognition specifically refers to the ability of
9. Retrieval specifically refers to the ability of
10. Semantic memory primarily stores information about
11. Altruism is a form of prosocial behavior that is motivated by
12. Conformity refers to the
13. Empathy refers to the ability to
14. If group members modify their opinions to align with a perceived group consensus, this is an example of
15. A set of group expectations for appropriate thoughts and behaviors of its members is called
16. The emotional bond between an infant and parent that affects the infant’s sense of security is
17. Cognitive development primarily concerns the strengthening of
18. The idea that even if something is out of sight, it still exists is called
19. An example of the sensitive period is the
20. Temperament is thought of as
21. Binocular vision requires
22. A blind spot is understood to be
23. An example of a gestalt principle is the
24. Inattentional blindness is thought of as
25. Perceptual constancy of shapes, brightness and size refers to the
26. Associative learning occurs when an individual
27. When a stimulus or experience occurs before a behavior that it gets paired with what occurs is
28. Observational learning is thought to largely derive from
29. Operant conditioning is a form of learning where
30. Taking away a pleasant stimulus to stop a behavior is an example of
31. The ability to self-monitor in social situations will especially depend on the
32. A lesion to the hippocampus would render an individual entirely unable to
33. Associative learning mostly relies on
34. What is likely to play the largest role in determining recognition performance on a cognitive task?
35. What kind of memory is impacted by infantile amnesia?
36. Social norms will typically be stored in
37. The development of secure attachment will crucially depend on the caregiver demonstrating high levels of
